# Genome‐Wide Meta‐Analysis Identifies Five Single‐Nucleotide Polymorphisms Associated With Both Vitiligo and Freckles in the Chinese Han Population

**DOI:** 10.1111/jocd.70342

**Published:** 2025-07-14

**Authors:** Jiayi Lu, Yirui Wang, Zhuo Li, Jing Yu, Can Song, Minhao Wang, Sihao Yan, Juan Du, Chunmeng Liu, Xiaofen Guo, Xinyu Feng, Wanli Niu, Mingwei Lv, Weiwei Chen, Yang Han, Qi Zhen, Liangdan Sun

**Affiliations:** ^1^ Department of Dermatology The First Affiliated Hospital of Anhui Medical University Hefei China; ^2^ Key Laboratory of Dermatology (Anhui Medical University), Ministry of Education Hefei China; ^3^ School of Public Health North China University of Science and Technology Tangshan Hebei China; ^4^ School of Clinical Medicine North China University of Science and Technology Tangshan Hebei China; ^5^ School of Basic Medicine North China University of Science and Technology Tangshan Hebei China; ^6^ Health Science Center North China University of Science and Technology Tangshan China; ^7^ North China University of Science and Technology Affiliated Hospital Tangshan China; ^8^ Inflammation and Immune Diseases Laboratory of North China University of Science and Technology Tangshan China

**Keywords:** freckles, GWAS, meta‐analysis, MHC, vitiligo

## Abstract

**Background:**

Vitiligo and freckles are both pigmentary disorders of the skin. The high prevalence of these conditions also places substantial strain on social healthcare systems. While vitiligo shows genetic predisposition, freckles exhibit autosomal dominant inheritance. Both conditions are closely linked to the immune system, potentially involving immune irregularities and autoimmune deficiencies.

**Objective:**

This study aimed to investigate the correlation between vitiligo and freckles in relation to major histocompatibility complex (MHC) regions to better understand the genetic factors influencing these chronic pigmentation disorders.

**Methods:**

Data from 3315 vitiligo patients, 7168 healthy controls, 524 freckle patients, and 4280 healthy controls were collected. A genome‐wide association study (GWAS) utilizing the Infinium Global Screening Array‐24 v2.0 BeadChip (GSA) and MHC region imputation was conducted, followed by meta‐analysis.

**Results:**

Through stepwise conditional analysis, five single‐nucleotide polymorphisms (SNPs) associated with both vitiligo and freckles were identified. These genetic markers shed light on common susceptibility factors between the two diseases.

**Conclusion:**

The findings of this study provide insights into the genetic basis of vitiligo and freckles, suggesting potential associations and shared pathways. Understanding these genetic correlations can aid in disease diagnosis, prevention strategies, and further exploration of biological mechanisms underlying these pigmentation disorders.

**Abbreviations**
GWAS: genome‐wide association study
LD: linkage disequilibrium
MAF: minor allele frequency
MHC: major histocompatibility complex
QC: quality control
SNP: single‐nucleotide polymorphism

## 1 Introduction

Vitiligo is an autoimmune disease characterized by patchy depigmentation of the skin, with white patches appearing in any part of the body [1]. The disease has been documented as early as 3500 years ago and has always had a high incidence [2]. Nowadays, vitiligo afflicts nearly 0.5%–2% of the population in the world [3]. There is no significant difference in the incidence between different regions, genders, and ethnicities. Similar to vitiligo, freckling is also a pigmentary disease, but different from the absence of pigment in vitiligo, the clinical manifestation of freckling is pigmentation [4]. Freckles are clinically characterized by small yellowish brown or brown pigment spots (1‐3 mm), which tend to occur in exposed parts, such as the face, especially on the bridge of the nose and cheeks [5]. Freckles have a high incidence rate in the general population, estimated to range from 1.5% to 33% [6, 7]. Both vitiligo and freckles have caused a serious psychological burden to patients, and the high incidence of these diseases has also placed greater pressure on social health care. Therefore, for this chronic pigmentary disease, it is necessary for us to further understand its pathogenesis and genetic law, providing a new way for disease prevention and diagnosis.

In this study, we focus on the genetic and immune factors of vitiligo and freckles. Research has shown that vitiligo has a certain genetic tendency, and freckles are autosomal dominant inheritance [5, 8]. Both diseases play important roles in the pathogenesis of immune factors [9, 10]. Therefore, we focus our attention on the major histocompatibility complex (MHC) region, which is located on chromosome 6P21 and is considered the most polymorphic human genetic system and is closely related to multiple immune mechanisms. Approximately 4 Mb, divided into sub‐regions class I (human leukocyte antigen [HLA]‐A, B, C), class II (HLA‐DR, DP, DQ), and class III (C2) [11]. In previous studies, we always analyzed the association of the MHC region with a certain disease, such as HLA and psoriasis, HLA and diabetes, HLA and tumor, and so on [12, 13, 14]. We are exploring for the first time the association between two distinct chronic pigment disorders and the MHC region.

We first collected case cohorts of vitiligo and freckles, sequenced them using Infinium Global Screening Array‐24 v2.0 BeadChip (GSA) chips, and inferred the MHC region. Then, through meta‐analysis, we analyzed the information inferred from the two groups of MHCs to identify the genetic association between these two chronic pigmentary diseases. Through this approach, we found that these two diseases have more than ten common amino acid mutations and single‐nucleotide polymorphisms (SNPs). This helps us further understand the genetic susceptibility factors of vitiligo and freckles, and their interrelationships.

## 2 Materials and Methods

### 2.1 Participants

This study included 524 patients with freckles, with 4280 healthy controls. There were 3315 vitiligo patients and 7168 healthy controls. Our healthy control sample was derived from previous studies [15]. The clinical diagnosis of all patients was confirmed by at least two experienced dermatologists. All controls were healthy individuals with no vitiligo or freckles, no other autoimmune disease or systemic disease, and no family history (including first‐, second‐, and third‐degree relatives). Clinical and demographic information was collected from cases and controls using the same structured questionnaire as previously [15]. Case and control groups were matched according to age, sex, and race. All participants received written informed consent.

### 2.2 Genotyping and Quality Control

Whole blood samples were subjected to DNA extraction by using the Flexi Gene DNA Extraction Kit (Qiagen). We used the GSA for sequencing analysis on both queue samples. We applied quality control (QC) procedures in the cohort by using PLINK 1.9 software to calculate genetic relationships in samples. Samples with (i) call rate < 98%, (ii) kinship, and (iii) heterozygosity > ±3 SD were filtered out. Exclusion criteria for genotyped SNPs were: (i) call rate < 95%; (ii) minor allele frequency (MAF) < 5%; and (iii) Hardy–Weinberg equilibrium with a *p* value ≤ 10^−4^ in controls.

### 2.3 HLA Imputation

Using the Han population reference panel as the reference database and Beagle 4.1 as the inference software14, the SNPs of the MHC region were imputed. The Chinese Han MHC database was used to determine the HLA types and amino acids of the MHC region. To improve inference speed, the large data sets were segmented and calculated in parallel with ten iterations and four threads for calculation. The fcGENE v1.7 software was used to convert between PLINK and Beagle formatted files. Beagle 4.1 software was used for imputation. Our team extracted HLA alleles and SNP data. Data with call rates ≤ 95% were excluded. We set the *r*2 value of > 0.3 for imputation dosage for SNPs; imputation dosage *r*2 > 0.3 for HLA amino acid and alleles; MAF < 0.05; and imputation quality (*r*2 > 0.3) for post‐imputation QC criteria.

### 2.4 Meta‐Analysis

To further explore the common loci associated with vitiligo and freckles susceptibility, we performed an inverse variance fixed‐effects meta‐analysis of weights proportional to the square root of the sample size based on the results of association analysis of two GWAS cohorts using METAL software (http://csg.sph.umich.edu/abecasis/Metal/index.html) [16]. We performed a traditional pairwise meta‐analysis for direct comparison. To ensure the accuracy of data, we used the *I* ^2^ statistic and set the inspection standard as < 30. The *p* value of the statistic was set at 1.83 × 10^−6^.

### 2.5 Stepwise Conditional Analysis

Our team conducted association analysis by using Plink2.0 software for stepwise conditional analysis. We applied conditional analysis by fixing one significant variant and stopping the procedure when no significant variant was observed.

## 3 Results

### 3.1 Sample Situation and Data Analysis

**FIGURE 1:**
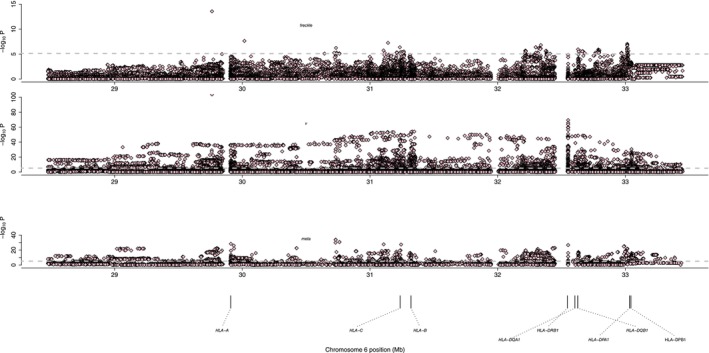
Process of MHC inference and meta‐analysis (V: Vitiligo): After conducting GAWS sequencing analysis on both queues, a Meta‐analysis of the sequencing data was conducted.

In order to further investigate the susceptibility genes and their loci of pigmentary diseases, we selected vitiligo and freckles for testing. After strict QC, we collected 524 patients with freckles, with 4280 healthy controls. There were 3315 vitiligo patients and 7168 healthy controls. We used the GSA chip for sequencing analysis on both queue samples. After conducting GAWS sequencing analysis on both queues, we conducted a meta‐analysis of the sequencing data. After meta‐analysis, we obtained a total of 171 SNPs with *p* values less than 1.26 × 10^−6^, which require further screening to explore their significance for the disease. The overall process of MHC inference and meta‐analysis is shown in Figure 1. We will introduce amino acid mutations and SNPs found by the GSA chip for vitiligo and freckles, respectively.

### 3.2 Vitiligo Site Information Detected by GSA Chip

We analyzed vitiligo with a new sample size using the GSA chip, and screened it with the condition of 1.26 × 10^−6^. We found 273 amino acid mutations, 415 SNPs, and 45 HLA typing remaining. Similar to previous studies, our findings suggest that mutations in the MHC region of vitiligo exist in multiple HLA genes, including but not limited to HLA‐A, HLA‐DRB1, HLA‐B, HLA‐C, and so on. Our study once again confirmed the important role of the MHC region in vitiligo, indicating the genetic diversity of vitiligo.

### 3.3 Freckles Site Information Detected by GSA Chip

We also analyzed the sample data of freckles using GSA chips, and then we once again verified the importance of the MHC region in freckle genetics. We still screened at 1.26 × 10^−6^ and obtained a total of 28 amino acid mutations, 66 SNP loci, and 5 HLA typings. Similar to vitiligo, these mutations are distributed in HLA‐A, HLA‐B, HLA‐C, HLA‐DQB1, and so on. Previous studies have rarely associated freckles with immunity. However, this study confirms the strong correlation between immunity and freckles through genetic factors. Therefore, further analysis is necessary. Do different chronic pigmentary diseases have certain immunological commonalities?

### 3.4 Amino Acid Mutations Associated With Pigmentary Diseases Revealed by a Meta‐Analysis

We classified and analyzed 171 SNPs, first identifying amino acid mutations. To prevent a certain site from having a significant impact on a certain disease and resulting in outstanding performance in meta‐analysis, we reviewed all sites and obtained 13 amino acid mutations in the MHC region (Table 1). We found that amino acid mutations related to diseases cover three genes: HLA‐A, HLA‐C, and HLA‐RB1. There is an interesting phenomenon that different amino acid mutations at the same amino acid site have different effects on diseases. For example, the HLA‐A156 amino acid is negatively correlated with the disease if it is leucine, but positively correlated with the disease if it is glutamine. Due to different amino acid mutations at the same gene locus, it is common to have different effects on diseases. In our meta‐analysis of vitiligo and freckles, we found 5 pairs, with the other 4 pairs being HLA‐C* 304 valine and methionine; serine and proline of HLA‐A* 105; threonine and arginine in HLA‐A* 163; aspartate and alanine in HLA‐A* 90. Due to the close relationship between skin pigments and immunity, metabolism, mental stress, and so on, it is likely that different amino acids affect the protein structure, leading to its progression toward different aspects of the disease.

**TABLE 1:** Meta‐analysis: Amino acid mutations in the MHC region.

Gene name	AA changes	SE	*p*	Direction
HLA‐DRB1*67	*I*	*0.0273*	2.05E‐27	‐‐
HLA‐C*77	—	0.0312	2.50E‐20	++
HLA‐C*88	—	0.0312	2.50E‐20	++
HLA‐A*156	L	0.0272	7.12E‐20	‐‐
HLA‐304	V	0.0289	3.51E‐14	++
HLA‐304	M	0.0289	3.51E‐14	‐‐
HLA‐A*156	Q	0.0283	7.51E‐13	++
HLA‐A*105	S	0.0299	8.03E‐12	‐‐
HLA‐A*105	P	0.0299	8.03E‐12	++
HLA‐A*163	T	0.0302	1.04E‐10	‐‐
HLA‐A*163	R	0.0302	1.04E‐10	++
HLA‐A*90	D	0.0302	1.18E‐10	++
HLA‐A*90	A	0.0302	1.18E‐10	‐‐

*Note:* Meta‐analysis revealing 13 amino acid mutations linked to pigmentary disorders.

Abbreviation: AA changes, Amino acid mutations.

### 3.5 Single‐Nucleotide Polymorphism Associated With Pigmentary Diseases Revealed by a Meta‐Analysis

We screened SNPs using the same method as amino acid mutations and found 15 SNP sites with *p* values less than 1.26 × 10^−6^. These SNPs are distributed in four genes, namely, HLA‐A, HLA‐C, HLA‐DRB1, and HLA‐DQB1. It is interesting that the rs9260156 mutation at the same HLA‐A gene locus has different effects on the disease. For example, if it is an A/T mutation at this locus, the impact on the disease is negative, and if it is an A/− mutation, it is positive. Among all SNPs, only this site exhibits this phenomenon. This SNP is located in the coding region, and mutations may have biological functional effects, such as noncoding script exit variant, missense variant, regulatory region variant, and so on. Through this meta‐analysis, independent loci were also aggregated into Table 2.

**TABLE 2:** Meta‐analysis: Single‐nucleotide polymorphism in the MHC region.

SNP	Allele	Position	Direction	*p*	GENE
rs17886918	A/T	32 551 970	‐‐	2.05E‐27	HLA‐DRB1
rs17408553	T/G	31 239 407	++	2.50E‐20	HLA‐C
rs2308557	T/C	31 239 417	++	2.50E‐20	HLA‐C
rs9260156	A/T	29 911 240	‐‐	7.12E‐20	HLA‐A
rs9256983	A/C	29 911 228	++	1.70E‐14	HLA‐A
rs1050326	C/G	31 238 147	‐‐	1.70E‐14	HLA‐C
rs1354654935	T/C	31 237 776	++	3.51E‐14	HLA‐C
rs9260156	A/−	29 911 240	++	7.51E‐13	HLA‐A
rs1136700	T/C	29 911 086	++	8.03E‐12	HLA‐A
rs9260155	T/C	29 911 239	++	8.61E‐11	HLA‐A
rs543623321	A/C	29 911 260	++	1.04E‐10	HLA‐A
rs3129017	C/G	29 911 261	++	1.04E‐10	HLA‐A
rs1136692	A/C	29 910 801	‐‐	1.18E‐10	HLA‐A
rs1632885	T/G	29 912 510	++	1.50E‐10	HLA‐A
rs1049130	A/G	32 629 859	++	3.59E‐10	HLA‐DQB1

*Note:* Meta‐analysis revealing 15 single‐nucleotide polymorphism sites with *p* values less than 1.26 × 10^−6^ linked to pigmentary disorders.

### 3.6 Results of Stepwise Conditional Regression Analyzed Using the Meta‐Analysis Data

In the above, we have conducted stable nuclear variant (SNV), HLA region type, and amino acid inference, and meta‐analysis of vitiligo and freckles using the GSA chip. The most significant locus was rs17886918 (*p* = 2.05E‐27). After conditioning this locus, we found that five independent loci were significant; among them, rs9260156 in the HLA gene was the most significant (*p* = 8.30E‐11; OR = 0.8293), and rs9260156; rs17408553; rs9256983; rs1136700; and rs2308557. Adding rs9260156 to the covariates for logistic regression analysis, the most significant two loci remained rs17408553 and rs2308557, while the remaining loci were no longer significant, and no new significant loci were found. Adding the loci found above one by one to the covariates for logistic regression analysis, no loci were significant. We have summarized the results of the association analysis of pigmentary diseases in Table 3.

**TABLE 3:** Stepwise analysis after adjusting.

STEP	Variant	OR	*p*	SE	L95	U95	Gene annotation
1	rs17886918	NA	NA	NA	NA	NA	*HLA‐DRB1*
2	rs9260156	0.8293	8.30E‐11	0.02882	0.7837	0.8775	*HLA‐A*
2	rs17408553	1.24	1.47E‐10	0.03352	1.161	1.324	*HLA‐C*
2	rs9256983	0.8401	9.80E‐09	0.03039	0.7915	0.8916	*HLA‐A*
2	rs1136700	0.8556	6.68E‐07	0.03137	0.8046	0.9099	*HLA‐A*
2	rs2308557	1.24	1.47E‐10	0.03352	1.161	1.324	*HLA‐C*

*Note:* Stepwise conditional analysis identified five SNPs linked to both vitiligo and freckles.

## 4 Discussion

Vitiligo and freckles are two opposite acquired pathologic conditions associated with skin pigmentation. However, they may have the same specific genetic background. Previous studies have indicated that the HLA region may be linked to genetic susceptibility to both vitiligo and freckles [17, 18]. Meta‐analyses have strongly suggested that HLA‐A2 is associated with vitiligo [17]. Among HLA Class II alleles, DRB1*07 has been significantly positively correlated with vitiligo in the Chinese Han population, with research confirming significant clinical differences between DRB1*07 positive and DRB1*07 negative patients [19]. While HLA associations have been established for vitiligo, their role in freckles remains less explored. Our study reaffirms previous reports linking HLA‐A, HLA‐C, and HLA‐DRB1 to vitiligo [20], and further validates the association of HLA‐C with freckles. Notably, we identify HLA‐A and HLA‐DRB1 as potential susceptibility loci for freckles.

These alleles also show pleiotropic effects. HLA‐A has been associated with metastatic uveal melanoma and has been reported as a susceptible allele in noncarriers of Thai Bessai disease [21, 22]. Additionally, HLA‐A is associated with multiple sclerosis, HBV infection of Han adult acute liver disease, and many other diseases [23, 24]. HLA‐C has been associated with several disease susceptibility genes, including inflammatory arthropathy [25], chronic obstructive pulmonary disease [26], inflammatory bowel disease [27], and so on. Previous research has also shown that expression of HLA‐DRB1 is correlated with the severity of IgAN and other diseases [28], suggesting broad immunogenetic roles.

Although the study provides insights into HLA variants for vitiligo and freckles, it has limitations. Due to extensive linkage disequilibrium (LD), high polymorphism, and strong genetic heterogeneity in the MHC region, we were unable to capture all genetic variants. Meanwhile, the effects of non‐HLA genes and noncoding variants remain to be studied. Despite the large sample size, limited queue independence necessitates further studies to clarify the common genetic structure of the vitiligo and freckle MHC regions and the exact role of the identified variants.

Our association and conditional analysis identified rs17886918 as the most significant SNP associated with vitiligo and freckle (*p* = 2.05E‐27). We are the first to combine the two pigmented diseases of vitiligo and freckles for a correlation analysis. No prior reports have established an association between rs17886918 and vitiligo or freckles in Chinese or other ethnic groups.

## 5 Conclusion

Our study complements the genetic interpretation of existing disease susceptibility genes, identifies common genetic structures in freckles and vitiligo that may contribute to both conditions, and may advance our understanding of HLA coding variants and the genetic structure of the MHC region. These new findings may enable subsequent analysis of biological pathways to identify new therapeutic targets that could improve patients' quality of life.

## Author Contributions

L.D.S., Q.Z., Y.H., and W.C. designed this project and the scientific objectives, conceptualized the study, revised the manuscript, and approved this paper. J.L., Y.R.W., and Z.L. analyzed the data and wrote the paper. J.Y., C.S., M.W., S.H.Y., J.D., C.L., X.G., X.Y.F., W.N., and MWL analyzed the data and organized pictures. All authors contributed to the paper.

## Ethics Statement

The authors confirm that the ethical policies of the journal, as noted on the journal's author guidelines page, have been adhered to, and the appropriate ethical review committee approval has been received. The study involved human skin biopsies and was conducted according to the guidelines of the Declaration of Helsinki, and approved by the Ethics Committee for Clinical Medical Research in the first affiliated Hospital of Anhui Medical University (consent number: 2023317).

## Conflicts of Interest

The authors declare no conflicts of interest.

## Data Availability

The data that support the findings of this study are available on request from the corresponding author. The data are not publicly available due to privacy or ethical restrictions.

## References

[1] M. Rodrigues , K. Ezzedine , I. Hamzavi , A. G. Pandya , and J. E. Harris , “New Discoveries in the Pathogenesis and Classification of Vitiligo,” Journal of the American Academy of Dermatology 77, no. 1 (2017): 1–13, 10.1016/j.jaad.2016.10.048. 28619550

[2] S. Barman , “Switra and Its Treatment in Veda,” Ancient Science of Life 15, no. 1 (1995): 71–74. 22556723 PMC3331180

[3] Y. Zhang , Y. Cai , M. Shi , et al., “The Prevalence of Vitiligo: A Meta‐Analysis,” PLoS One 11, no. 9 (2016): e0163806, 10.1371/journal.pone.0163806. 27673680 PMC5038943

[4] J. McKesey , A. Tovar‐Garza , and A. G. Pandya , “Melasma Treatment: An Evidence‐Based Review,” American Journal of Clinical Dermatology 21, no. 2 (2020): 173–225, 10.1007/s40257-019-00488-w. 31802394

[5] T. Passeron and M. Picardo , “Melasma, a Photoaging Disorder,” Pigment Cell & Melanoma Research 31, no. 4 (2018): 461–465, 10.1111/pcmr.12684. 29285880

[6] D. I. McLean and R. P. Gallagher , ““Sunburn” Freckles, Café‐Au‐Lait Macules, and Other Pigmented Lesions of Schoolchildren: The Vancouver Mole Study,” Journal of the American Academy of Dermatology 32, no. 4 (1995): 565–570. 7896944 10.1016/0190-9622(95)90338-0

[7] K. D. Werlinger , I. L. Guevara , C. M. González , et al., “Prevalence of Self‐Diagnosed Melasma Among Premenopausal Latino Women in Dallas and Fort Worth, Tex,” Archives of Dermatology 143, no. 3 (2007): 424–425. 17372115 10.1001/archderm.143.3.424

[8] T. Passeron and J.‐P. Ortonne , “Physiopathology and Genetics of Vitiligo,” Journal of Autoimmunity 25, no. Suppl (2005): 63–68. 10.1016/j.jaut.2005.10.001 16298511

[9] E. Roh , J.‐E. Kim , J. Y. Kwon , et al., “Molecular Mechanisms of Green Tea Polyphenols With Protective Effects Against Skin Photoaging,” Critical Reviews in Food Science and Nutrition 57, no. 8 (2017): 1631–1637, 10.1080/10408398.2014.1003365. 26114360

[10] J. Chen , S. Li , and C. Li , “Mechanisms of Melanocyte Death in Vitiligo,” Medicinal Research Reviews 41, no. 2 (2021): 1138–1166, 10.1002/med.21754. 33200838 PMC7983894

[11] J. Trowsdale and J. C. Knight , “Major Histocompatibility Complex Genomics and Human Disease,” Annual Review of Genomics and Human Genetics 14 (2013): 301–323, 10.1146/annurev-genom-091212-153455. PMC4426292 23875801

[12] H.‐F. Zheng , X.‐B. Zuo , W.‐S. Lu , et al., “Variants in MHC, LCE and IL12B Have Epistatic Effects on Psoriasis Risk in Chinese Population,” Journal of Dermatological Science 61, no. 2 (2011): 124–128, 10.1016/j.jdermsci.2010.12.001. 21208785

[13] R. Mishra , M. Åkerlund , D. L. Cousminer , et al., “Genetic Discrimination Between LADA and Childhood‐Onset Type 1 Diabetes Within the MHC,” Diabetes Care 43, no. 2 (2020): 418–425, 10.2337/dc19-0986. 31843946 PMC6971787

[14] K. Yamamoto , A. Venida , J. Yano , et al., “Autophagy Promotes Immune Evasion of Pancreatic Cancer by Degrading MHC‐I,” Nature 581, no. 7806 (2020): 100–105, 10.1038/s41586-020-2229-5. 32376951 PMC7296553

[15] W. Chen , W. Wang , L. Yong , et al., “Genome‐Wide Meta‐Analysis Identifies Ten New Psoriasis Susceptibility Loci in the Chinese Population,” Journal of Genetics and Genomics 49, no. 2 (2022): 4. 10.1016/j.jgg.2021.10.001 34695602

[16] C. J. Willer , L. Yun , and G. R. Abecasis , “METAL: Fast and Efficient Meta‐Analysis of Genomewide Association Scans,” Bioinformatics 26, no. 17 (2010): 2190–2191. 20616382 10.1093/bioinformatics/btq340 PMC2922887

[17] J. B. Liu , M. Li , H. Chen , et al., “Association of Vitiligo With HLA‐A2: A Meta‐Analysis,” Journal of the European Academy of Dermatology and Venereology 21, no. 2 (2007): 205–213. 17243956 10.1111/j.1468-3083.2006.01899.x

[18] V. Laville , S. L. Clerc , K. Ezzedine , et al., “A Genome‐Wide Association Study in Caucasian Women Suggests the Involvement of HLA Genes in the Severity of Facial Solar Lentigines,” Pigment Cell & Melanoma Research 29, no. 5 (2016): 550–558, 10.1111/pcmr.12502. 27327535

[19] D. Y. Hu , Y. Q. Ren , K. J. Zhu , et al., “Comparisons of Clinical Features of HLA‐DRB1*07 Positive and Negative Vitiligo Patients in Chinese Han Population,” Journal of the European Academy of Dermatology and Venereology 25, no. 11 (2011): 1299–1303, 10.1111/j.1468-3083.2010.03971.x. 21241376

[20] C. Quan , Y.‐Q. Ren , L.‐H. Xiang , et al., “Genome‐Wide Association Study for Vitiligo Identifies Susceptibility Loci at 6q27 and the MHC,” Nature Genetics 42, no. 7 (2010): 614–618, 10.1038/ng.603. 20526339

[21] L. N. Chen and R. D. Carvajal , “Tebentafusp for the Treatment of HLA‐A*02:01‐Positive Adult Patients With Unresectable or Metastatic Uveal Melanoma,” Expert Review of Anticancer Therapy 22, no. 10 (2022): 1017–1027, 10.1080/14737140.2022.2124971. 36102132 PMC10184536

[22] W. Louthrenoo , N. Kasitanon , K. Pathanapitoon , et al., “Contribution of HLA‐B*51:01 and ‐A*26:01 to Behçet's Disease and Their Clinical Association in Thai Patients,” International Journal of Rheumatic Diseases 23, no. 2 (2020): 247–255, 10.1111/1756-185X.13785. 31944588

[23] Z. Ghobadi , K. Mahnam , and M. Shakhsi‐Niaei , “In‐Silico Design of Peptides for Inhibition of HLA‐A*03‐KLIETYFSK Complex as a New Drug Design for Treatment of Multiples Sclerosis Disease,” Journal of Molecular Graphics & Modelling 111 (2022): 108079, 10.1016/j.jmgm.2021.108079. 34837787

[24] D. Li , Z. Zeng , S. Zhao , et al., “Human Leukocyte Antigen Polymorphism HLA‐A*24:02 Is Associated With Acute Liver Disease in HBV‐Infected Han Chinese Adults,” Immunological Investigations 52, no. 6 (2023): 767–778, 10.1080/08820139.2023.2232409. 37417317

[25] J. Roudier , E. Massy , and N. Balandraud , “Diagnostic Contribution of HLA‐A,B,C,DR Genotyping in Inflammatory Joint Disease,” Joint, Bone, Spine 85, no. 5 (2018): 511–513, 10.1016/j.jbspin.2018.02.007. 29654946

[26] T. Mkorombindo , T. K. Tran‐Nguyen , K. Yuan , et al., “HLA‐C and KIR Permutations Influence Chronic Obstructive Pulmonary Disease Risk,” JCI Insight 6, no. 19 (2021): e150187, 10.1172/jci.insight.150187. 34464355 PMC8525585

[27] P. Castro‐Santos , M. A. Moro‐García , R. Marcos‐Fernández , R. Alonso‐Arias , and R. Díaz‐Peña , “ERAP1 and HLA‐C Interaction in Inflammatory Bowel Disease in the Spanish Population,” Innate Immunity 23, no. 5 (2017): 476–481, 10.1177/1753425917716527. 28651467

[28] X. Zhan , F. Deng , A. Y. Wang , et al., “HLA‐DQB1 and HLA‐DRB1 Expression Is Associated With Disease Severity in IgAN,” Ann Palliat Med 10, no. 9 (2021): 9453–9466, 10.21037/apm-21-2065. 34628871

